# Measuring cannabis consumption: Psychometric properties of the Daily Sessions, Frequency, Age of Onset, and Quantity of Cannabis Use Inventory (DFAQ-CU)

**DOI:** 10.1371/journal.pone.0178194

**Published:** 2017-05-26

**Authors:** Carrie Cuttler, Alexander Spradlin

**Affiliations:** Department of Psychology, Washington State University, Pullman, WA, United States of America; Waseda University, JAPAN

## Abstract

**Objective:**

We created the Daily Sessions, Frequency, Age of Onset, and Quantity of Cannabis Use Inventory (DFAQ-CU) because the current lack of psychometrically sound inventories for measuring these dimensions of cannabis use has impeded research on the effects of cannabis in humans.

**Method:**

A sample of 2,062 cannabis users completed the DFAQ-CU and was used to assess the DFAQ-CU’s factor structure and reliability. To assess validity, a subsample of 645 participants completed additional measures of cannabis dependence and problems (Marijuana Smoking History Questionnaire [MSHQ], Timeline Followback [TLFB], Cannabis Abuse Screening Test [CAST], Cannabis Use Disorders Identification Test Revised [CUDIT-R], Cannabis Use Problems Identification Test [CUPIT], and Alcohol Use Disorder Identification Test [AUDIT]).

**Results:**

A six-factor structure was revealed, with factors measuring: daily sessions, frequency, age of onset, marijuana quantity, cannabis concentrate quantity, and edibles quantity. The factors were reliable, with Cronbach’s alpha coefficients ranging from .69 (daily sessions) to .95 (frequency). Results further provided evidence for the factors’ convergent (MSHQ, TLFB), predictive (CAST, CUDIT-R, CUPIT), and discriminant validity (AUDIT).

**Conclusions:**

The DFAQ-CU is the first psychometrically sound inventory for measuring frequency, age of onset, and quantity of cannabis use. It contains pictures of marijuana to facilitate the measurement of quantity of marijuana used, as well as questions to assess the use of different forms of cannabis (e.g., concentrates, edibles), methods of administering cannabis (e.g., joints, hand pipes, vaporizers), and typical THC levels. As such, the DFAQ-CU should help facilitate research on frequency, quantity, and age of onset of cannabis use.

## Introduction

A growing number of states are moving toward the legalization of cannabis for medical and recreational purposes. Consequently, perceived risks and stigma surrounding use are declining [1, 2], and the percentage of the population using cannabis is expanding [3]. This is creating a sense of urgency to intensify research on the effects of cannabis in humans. One impediment to such efforts is an absence of psychometrically sound self-report inventories for measuring frequency, quantity, and age of onset of cannabis use.

Most investigations of the chronic or residual effects of cannabis in humans have employed valid and reliable measures of cannabis use disorders (e.g. Cannabis Abuse Screening Test [CAST], Cannabis Use Disorders Identification Test Revised [CUDIT-R]), and problems associated with use (e.g., Cannabis Use Problems Identification Test [CUPIT]). Annaheim [4] recently published a review of 44 instruments designed to assess cannabis-related problems and concluded that the CAST, CUDIT-R, and CUPIT are the most appropriate inventories for screening cannabis-related problems. Similarly, López-Pelayo and colleagues [5] conducted a meta-analytic review that identified 25 instruments to assess cannabis use and cannabis-related problems. They also identified the CAST and CUDIT as two of the highest performing inventories for assessing cannabis-related problems.

In contrast to the abundance of inventories for assessing cannabis-related problems, there is a paucity of inventories for measuring frequency, quantity, and age of onset of cannabis use. Due to the absence of psychometrically sound inventories, most researchers rely on in-house survey questions to assess these aspects of cannabis use. While these questions typically have good face validity, they vary substantially across studies, and they lack information pertaining to validity and reliability. The use of such questions therefore lacks the necessary scientific rigor we need to progress this field of research and hinders the ability to make comparisons across studies.

Other investigators have borrowed items from national surveys (e.g., the National Epidemiological Survey on Alcohol and Related Conditions, the Epidemiologic Catchment Area Study, the National Comorbidity Survey, the National Survey on Drug Use and Health) to measure frequency and age of onset of cannabis use. This represents an improvement upon the reliance on in-house survey questions because it permits for comparisons across studies using the same items. However, these national surveys were not developed as comprehensive assessments of cannabis consumption. As such they typically assess only one or two aspects of cannabis use (e.g., frequency, age of onset), and neglect other potentially important aspects of cannabis consumption (e.g., quantity of use).

The Timeline Followback Method for Marijuana (TLFB) [6] is a popular, valid, and reliable method of assessing recent cannabis consumption [7, 8]. To assess frequency of use, participants are shown a calendar of the last 30 days and are instructed to mark all of the days that they smoked joints. To obtain a measure of quantity of cannabis used, participants can be further instructed to indicate the number of joints they smoked on each day. In addition to being taxing for participants, this method may be subject to retrospective recall bias that likely varies as a function of the frequency or regularity of use (e.g., individuals who use cannabis on a daily basis would likely have an easier time accurately completing the calendar than individuals who engage in sporadic use). Moreover, the measurement of quantity of use with “number of joints smoked” is problematic because neither the size of the joints nor the potency are considered and because it disregards the fact that cannabis is often ingested using other methods (e.g., bongs, vaporizers, edibles). Indeed, only 10% of our sample reported using joints as their primary method of cannabis consumption. This method also focuses only on recent use and does not permit for the assessment of duration or age of onset of use. Finally, the TLFB is typically administered via interview [8], which further adds to the taxing nature of the measure.

To our knowledge, the Marijuana Smoking History Questionnaire (MSHQ) [9] is the only published self-report inventory for measuring all three aspects of cannabis consumption: frequency, quantity, and age of onset. The MSHQ has a number of strengths. First, it uses visually depicted diagrams of various sized joints to aid the measurement of quantity of marijuana used. Second, it assesses various methods of ingesting cannabis (joints, bowls, bongs, one-hitters, food). Third, it assesses the typical context of use (alone or with others). Finally, it is one of the only published inventories to include questions assessing age of onset of cannabis use. Unfortunately, the MSHQ also has a number of limitations. First, the images used to depict joints are rather crude, pixelated drawings that don’t closely resemble real joints. Moreover, there are no depictions of marijuana prepared for other methods of administration (i.e., pictures of marijuana in bud form or loose form). Second, the assessment of frequency relies on rating scales with only the endpoints (and in one case, a midpoint) of the scale labeled, which introduces unnecessary subjectivity in responses. Third, in assessing the age that regular use started, the definition of regular use is not provided, which introduces additional random variability in responses. Fourth, there are no standard published scoring procedures. Fifth, and of greatest concern, is that, to our knowledge, the psychometric properties of the MSHQ have not been assessed nor published. Therefore, the factor structure, reliability, and validity of the questionnaire are entirely unknown.

The glaring absence of psychometrically-evaluated inventories for measuring frequency, quantity, and age of onset of cannabis use, and the limitations of the existing inventories, motivated us to create a new inventory, which we named the Daily Sessions, Frequency, Age of Onset, and Quantity of Cannabis-Use Inventory (DFAQ-CU). To our knowledge, the DFAQ-CU is the first measure to include a picture of different quantities (⅛ gram, ¼ gram, ½ gram, ¾ gram, 1 gram) of actual marijuana in bud, loose, and joint form to facilitate the identification of the quantity of cannabis typically used. The DFAQ-CU also measures a variety of different methods of administering cannabis (joints, blunts, bongs, hand pipes, vaporizers, hookahs, edibles), amounts personally used (as opposed to shared), and typical THC levels in cannabis used. Further, it includes a number of optional screening questions (e.g., “How high are you right now?”) to offer researchers a means to screen and/or further characterize their sample.

To our knowledge, the DFAQ-CU is also the first cannabis use inventory to measure the use of cannabis concentrates (e.g. oil, wax, shatter). Concentrates are becoming increasingly popular, especially in states that have legalized recreational and/or medical cannabis use [10], and there is elevating concern that the detrimental effects of, and tolerance to, cannabis in concentrated form will be magnified due to the extremely high levels of THC they contain [11]. However, due to their recent development and the lack of published inventories for measuring the use of concentrates, their actual effects are largely unknown.

The present study was conducted to examine the psychometric properties of our newly developed inventory for measuring various aspects of cannabis consumption, including frequency, quantity, and age of onset of cannabis use. Specifically, the present study was conducted to assess the factor structure, reliability, and validity of the DFAQ-CU.

## Method

### Procedure

The Washington State University Institutional Review Board approved the studies [Approval Numbers: 14185–005, 15110, 14183]. All participants provided informed consent by reading the written consent form and then clicking ‘I agree’ to indicate voluntary consent to participate. Participants were assured that their responses would be kept confidential. Only the principal investigator and co-investigator for the approved studies had access to the names of participants and the password-protected data files. All survey responses were identified with only a unique ID code. After providing informed consent, participants completed an online survey that contained the DFAQ-CU and was designed to assess correlates of cannabis use. Participation required approximately one hour, and participants were compensated with course credit.

### Participants

A sample of 2,630 undergraduate students completed the DFAQ-CU. These participants were recruited from the Psychology Subject Pool at a major university in Washington State. This is a system that provides students, who are enrolled in eligible psychology courses, with the opportunity to participate in research studies for extra credit. They are able to choose from a large number of different studies to participate in. The study description and consent form indicated that the study was designed to assess various measures of cannabis consumption and correlates of cannabis use. Data collection took place between September 2015 and May 2016.

The 10-item deviant responding validity subscale of the Psychopathic Personality Inventory (PPI) [12] was interspersed throughout the surveys to detect random responders. In total 176 participants (7% of the sample) were deemed random responders and were excluded. An additional 392 participants (15% of the sample) were excluded because they had never used cannabis. The total eligible sample contained 2,062 cannabis users. A subset of the total sample, comprising 645 cannabis users, completed additional measures of cannabis and alcohol use described in the Measures section. This subset was also recruited from the Psychology Subject Pool. The basic demographic characteristics and cannabis use patterns of both samples are displayed in Table 1. As shown in this table, the characteristics of these two samples did not vary substantially.

**Table 1:** Sample characteristics.

Total Sample (N = 2062)		Subsample (*n* = 645)	
**Demographics**		**Demographics**	
Age	*M* = 20.15 (*SD* = 2.28) Range = 18–46	Age	*M* = 20.07 (*SD* = 2.14) Range = 18–36
Sex	Female 69.8%	Sex	Female 66.6%
	Male 30.2%		Male 33.4%
Ethnicity	White 69.4%	Ethnicity	White 69.0%
	Hispanic/Latino 10.6%		Hispanic/Latino 9.9%
	Black 6.9%		Black 7.3%
	Asian 4.7%		Asian 5.1%
	Other 3.6%		Other 3.4%
	Pacific Islander 3.2%		Pacific Islander 3.7%
	Native American 1.6%		Native American 1.5%
**Cannabis Use Patterns**		**Cannabis Use Patterns**	
Use in Past Month Among All Users	68.0%; *M* = 7.68 days; (*SD* = 10.14)	Use in Past Month Among All Users	76.0% *M* = 8.44 days; (*SD* = 10.42)
Use in Past Week Among All Users	52.3%; *M* = 1.82 days; (*SD* = 2.40)	Use in Past Week Among All Users	57.8% *M* = 1.99 days; (*SD* = 2.44)
Grams of Marijuana Per Day Among Marijuana Users	*M* = 0.99 (*SD* = 2.25)	Grams of Marijuana Per Day Among Marijuana Users	*M* = 1.32 (*SD* = 2.72)
Hits of Concentrates in Typical Day of Use Among Concentrate Users	*M* = 3.82 (*SD* = 3.62)	Hits of Concentrates in Typical Day of Use Among Concentrate users	*M* = 3.82 (*SD* = 3.85)
Typical Mgs of THC in Edibles Among Edible Users	*M =* 6.57 (*SD* = 2.63)	Typical Mgs of THC in Edibles Among Edible Users	*M =* 6.65 (*SD* = 2.65)
# Marijuana Sessions Per Day Among Marijuana Users	*M* = 1.41 (*SD* = 1.29)	# Marijuana Sessions Per Day Among Marijuana Users	*M* = 1.49 (*SD* = 1.32)
# Concentrate Sessions Per Day Among Concentrate Users	*M* = 1.62 (*SD* = 1.16)	# Concentrate Sessions Per Day Among Concentrate Users	*M* = 1.53 (*SD* = 1.10)
Marijuana % THC	*Mdn* = 15–19%	Marijuana % THC	*Mdn* = 15–19%
Concentrates % THC	*Mdn* = 70–79%	Concentrates % THC	*Mdn* = 70–79%
Age First Tried Among All Users	*M* = 16.58 (*SD* = 2.30)	Age First Tried Among All Users	*M* = 16.70 (*SD* = 2.34)
Primary Method	Bong– 37.7%	Primary Method	Bong– 39.2%
	Hand Pipe– 25.0%		Hand Pipe– 29.1%
	Joints– 10.4%		Joints– 9.6%
	Blunts– 5.6%		Blunts– 5.4%
	Hookah– 0.8%		Hookah– 0.8%
	Vaporizer– 2.5%		Vaporizer– 3.6%
	Edibles– 6.1%		Edibles– 7.3%
	Other– 1.3%		Other– 1.6%
Primary Type	Marijuana– 80.4%	Primary Type	Marijuana– 86.2%
	Edibles– 5.7%		Edibles– 6.8%
	Concentrates– 4.5%		Concentrates– 4.8%
	Other– 0.1%		Other– 0.2%
Prescription for Use Among All Users	3.6%	Prescription for Use Among All Users	3.9%

### Measures

The total sample completed a short demographics questionnaire, the PPI items, and the DFAQ-CU. We initially developed the 41-item DFAQ-CU to measure frequency, age of onset, and quantity of cannabis used. We designed 11 items to measure frequency, four items to measure age of onset, and nine items to measure quantity of cannabis use. Therefore, there were a total of 24 core items designed to measure these three aspects of cannabis use. The three subscales were constructed such that higher scores would indicate more frequent use, older age of onset, and higher quantity of use. The age of onset items probe for age of first use, age of regular cannabis use, age of daily or near daily cannabis use, and frequency of cannabis use before the age of 16. In an attempt to capture a regular pattern of use that is not heavy use, regular use is defined in the questionnaire as use of cannabis two or more times per month for six months or longer [13, 14, 15]. The age of 16 was targeted to be consistent with previous research that has defined early/adolescent onset use at this age [15, 16, 17]. The remaining items are used to establish skip logic and to screen and characterize the sample. These screening/characterization items are further described in the Discussion section. The complete revised inventory with response options and scoring information are provided in the S1 File.

The subsample of 645 participants also completed an online self-report version of the TLFB for Marijuana [6] and the MSHQ [9]. Moreover, they completed the CAST [18], which measures risk of cannabis use disorder and dependence; the CUDIT-R [19], which measures cannabis misuse; the Marijuana Screening Inventory (MSI-X) [20], which measures adverse effects associated with cannabis use; the CUPIT [21], which measures risky and problematic cannabis use; and the Alcohol Use Disorder Identification Test (AUDIT) [22], which measures harmful alcohol use.

### Data treatment and analysis

All variables were screened for univariate outliers, defined as scores falling more than 3.29 standard deviations from the mean, and the small number of outlying variables detected (< 1%) were replaced with a score equivalent to 3.29 standard deviations from the mean [23]. Due to the large number of analyses and the large sample size, a conservative alpha of .01 was used to determine statistical significance. All analyses were conducted using IBM SPSS 24. The complete dataset is provided in the S2 File.

Before factor analyzing the DFAQ-CU, we examined the sphericity of the 24-items to be included using the Kaiser-Meyer-Olkin (KMO) measure of sampling adequacy [24, 25] and Bartlett’s test of sphericity [26, 27]. Results revealed a KMO of 0.77, and Barlett’s test was significant, indicating that the items were appropriate to factor analyze [28].

The 24 core items of the DFAQ-CU (i.e., those designed to measure frequency, age of onset, and quantity of cannabis use) were then analyzed using both principal axis factoring and maximum likelihood estimation with a direct oblimin-rotated solution. This oblique method of rotation was selected under the assumption that the factors would be correlated. Delta was set to 0, and pairwise deletion was used for cases with missing data. Following the Kaiser-Guttman rule, factors with an eigenvalue of 1 or higher [29] were extracted. The scree plot was also inspected to confirm that the slope of the eigenvalues leveled off after the same number of factors as those identified using the Kaiser-Guttman rule. Finally, a factor loading cut-off of .45 was employed [23, 30].

Prior to conducting the reliability and validity analyses, scores on each of the 24 core DFAQ-CU items were standardized by z-score transformation. This transformation was necessary because the response scales used for the DFAQ-CU items vary considerably. Factor scores were computed by calculating the mean of all of the z-transformed items that loaded on each factor. The mean was used so that missing data on individual items would not bias the scores. All DFAQ-CU factors except for the age of onset factor were coded such that higher scores indicate more cannabis use (e.g., higher scores on the daily session factor indicate more sessions of cannabis use per day, higher frequency scores indicate more frequent use, higher quantity scores indicate higher quantities used). The age of onset factor was coded such that higher scores indicate later age of onset of cannabis use.

Cronbach’s alpha was used to assess the internal consistency of each of the factors. The following guidelines were used to interpret the reliability coefficients: .90 and above, excellent; .80-.89, good; .70-.79, acceptable; .60-.69, questionable, .50-.59, poor; less than .50, unacceptable [31].

Validity for the DFAQ-CU was established by examining the correlations between each factor and scores on the additional measures of cannabis and alcohol use described in the Measures section. Cohen’s [32] guidelines of interpreting correlations of .10, .30, and .50, as small, medium, and large, respectively were used to interpret the validity coefficients. We relied on the TLFB and MSHQ to assess convergent validity. Although there are no scoring criteria published for the MSHQ, we first standardized scores on each item, and then we computed the mean of the three items that measure frequency of use to create a frequency subscale (items 2, 4, and 11), the mean of the two items that measure quantity of use to derive a quantity subscale (items 3 and 10), and the mean of the two items that measure age of onset of use to acquire an age of onset subscale (items 7 and 8).

## Results

### Factor analysis

As shown in Table 2, the factor analysis using principal axis factoring yielded a six-factor solution that retained 22 of the 24 core DFAQ-CU items. These factors accounted for approximately 77% of the available variance. The factors were identified as representing: frequency (nine items accounting for 37% of unique variance), age of onset (four items; 12% of unique variance), marijuana quantity (three items; 10% of unique variance), cannabis concentrate quantity (three items; 8% of unique variance), daily sessions (two items; 5% of unique variance), and edibles quantity (one item; 4% of unique variance). None of the 22 items cross-loaded (i.e., all had cross-factor loadings ≤ .45). Table 3 shows the factor correlations. The factor structure produced using maximum likelihood estimation was consistent with the pattern identified using principal axis factoring.

**Table 2:** Factors loadings for the 24 core items of the DFAQ-CU.

Items	Factors					
	Daily Sessions	Frequency	Age of Onset	Marijuana Quantity	Concentrate Quantity	Edible Quantity
On a typical day you use cannabis concentrates, how many sessions do you have?	**-.901**	-.062	.015	-.065	.102	-.101
On a typical day you use marijuana, how many sessions do you have?	**-.678**	.071	-.022	.237	-.018	.101
Which of the following best captures the average frequency you currently use cannabis?	-.046	**.940**	.020	-.003	-.018	-.034
Which of the following best captures when you last used cannabis?	.009	**.860**	.049	.009	-.016	-.002
Approximately how many days of the past month did you use cannabis?	-.047	**.846**	.023	.001	-.002	.125
Which of the following best captures your pattern of cannabis use throughout the week?	.034	**.842**	.040	.011	-.026	-.014
How many days of the past week did you use cannabis?	-.037	**.811**	.016	-.043	.017	.146
Which of the following best captures the number of times you have used cannabis in your entire life?	-.072	**.746**	-.234	.013	-.028	.033
How many hours after waking up do you typically first use cannabis?	-.231	**.566**	-.009	-.003	.070	.067
How many times a day, on a typical weekday, do you use cannabis?	-.439	**.464**	-.010	.026	.007	.189
How many times a day, on a typical weekend, do you use cannabis?	-.399	**.450**	.013	.068	.003	.265
How old were you when you FIRST STARTED using cannabis on a daily or near daily basis?	.016	.305	**.931**	.009	.007	-.056
How old were you when you FIRST STARTED using cannabis regularly (2 or more times/month)?	-.026	.181	**.892**	-.016	-.023	.000
How old were you when you FIRST tried cannabis?	-.014	-.220	**.691**	.037	.034	-.007
Which of the following best captures the average frequency that you used cannabis before the age of 16?	.022	-.209	**.673**	-.038	-.049	.002
On a typical day you use marijuana, how much do you personally use?	-.068	-.052	.004	**.978**	-.005	047
In a typical session, how much marijuana do you personally use?	-.027	-.103	-.008	**.943**	-.081	-.193
In a typical week you use marijuana, how much marijuana do you personally use?	.033	.110	-.009	**.650**	.134	.415
On a typical day you use cannabis concentrates, how many hits do you personally take?	-.141	-.045	.002	-.069	**.871**	.056
In a typical session you use cannabis concentrates, how many hits do you personally take?	.081	-.152	-.029	-.021	**.768**	.010
How many hits of cannabis concentrates did you personally take yesterday?	-.204	.145	.047	.032	**.512**	.037
When you eat edibles, how many milligrams of THC do you personally ingest in a typical session?	-.051	.112	-.098	-.030	.019	**.459**
In a typical week you use cannabis concentrates, how many grams do you personally use?	.019	.325	-.066	.186	.402	-.316
How much marijuana did you personally use yesterday?	.058	.321	.044	.307	.133	.337

*Note*. Minimum factor loading = .50.

**Table 3:** Correlations between the factors of the DFAQ-CU.

	Daily Sessions	Frequency	Age of Onset	Marijuana Quantity	Concentrate Quantity
Daily Sessions	—				
Frequency	.522**	—			
Age of Onset	-.118**	-.229**	—		
Marijuana Quantity	.274**	.246**	-.073	—	
Concentrate Quantity	.403**	.256**	-.142*	.181*	—
Edible Quantity	.183**	.363**	-.158**	.125	.091

Note.

* *p* < .01

** *p* < .001.

### Reliability analyses

The frequency factor showed excellent reliability (α = .95), the marijuana quantity and age of onset factors showed good reliability (α = .88 and α = .81), and the cannabis concentrate factor showed adequate reliability (α = .76). The two-item daily session factor showed questionable reliability (α = .69). Since only one item loaded on the edibles factor, internal consistency could not be computed.

### Validity analyses

#### Convergent validity

As shown in Table 4, the frequency factor of the DFAQ-CU demonstrated high convergent validity with the MSHQ frequency subscale we derived, as well as with the TLFB measure of number of days a joint was smoked. Similarly, the age of onset factor demonstrated high convergent validity with scores on the MSHQ age of onset subscale. As shown in Table 4, all three of the DFAQ-CU quantity factors showed small but significant correlations with the MSHQ quantity subscale, and the DFAQ-CU concentrate and marijuana quantity factors showed small but significant correlations with the TLFB mean number of joints smoked per day. Although there are no existing measures of daily sessions, the DFAQ-CU daily sessions factor was moderately correlated with the MSHQ frequency subscale.

**Table 4:** Convergent, predictive, and discriminant validity of the DFAQ-CU factors (n = 645).

Measures	DFAQ-CU Factors					
	Daily Sessions	Frequency	Age of Onset	Marijuana Quantity	Concentrate Quantity	Edible Quantity
**Convergent Validity**						
MSHQ Frequency	.495**	.856**	-.289**	.197**	.253*	.207*
MSHQ Quantity	.408**	.643**	-.230**	.252**	.275**	.221*
MSHQ Age of Onset	-.011	-.093	.638**	.018	.029	-.059
TLFB Days	.207**	.705**	-.082	.069	-.145	.274*
TLFB Joints Mean	.330**	.581**	-.173**	.171*	.227*	.167
**Predictive Validity**						
CAST	.379**	.713**	-.280**	.180*	.239*	.246*
CUDIT-R	.387**	.731**	-.226**	.198**	.187*	.224*
CUPIT	.468**	.849**	-.281**	.175*	.278**	.256**
MSI-X	.303**	.625**	-.265**	.172*	.225*	.297**
**Discriminant Validity**						
AUDIT	.069	.227**	-.267**	.060	.068	.295**

*Note*. AUDIT = Alcohol Use Disorders Identification Test; CAST = Cannabis Abuse Screening Test; CUDIT-R = Cannabis Use Disorders Identification Test–Revised; CUPIT = Cannabis Use Problems Identification Test; MSHQ = Marijuana Smoking History Questionnaire; MSI-X = Marijuana Screening Inventory; TLFB = Timeline Followback.

* *p* < .01

** *p* < .001.

#### Predictive validity

As shown in Table 4, the frequency factor demonstrated excellent ability to predict (i.e., high correlations with) cannabis use disorder symptoms and cannabis use problems (CAST, CUDIT-R, CUPIT, MSI-X). The daily sessions factor showed moderate-sized, significant correlations with the CAST, CUDIT-R, CUPIT, and MSI-X. The age of onset, marijuana quantity, and edibles quantity factors of the DFAQ-CU showed small but significant correlations with scores on these four inventories. Finally, the concentrate quantity factor demonstrated small but significant correlations with the CAST, CUPIT, and MSI-X.

#### Discriminant validity

The daily sessions, marijuana quantity, and concentrate quantity factors of the DFAQ-CU were unrelated to alcohol use (AUDIT scores). The frequency, age of onset, and edible quantity factors from the DFAQ-CU showed small but statistically significant correlations with the AUDIT, (see Table 4).

## Discussion

Frequency, age of onset, and quantity of cannabis use are increasingly being recognized as important variables to consider when examining the effects of cannabis in humans [33]. However, investigations into these variables have been hindered by a lack of psychometrically sound inventories for their measurement. The present study was conducted to fill this gap; specifically, to assess the psychometric properties of a new inventory that was developed to measure frequency, age of onset, and quantity of cannabis used.

The DFAQ-CU was originally designed as a 41-item inventory. Twenty-four core items were developed specifically to measure frequency, age of onset, and quantity of cannabis use. A factor analysis revealed that the DFAQ-CU comprises six factors measuring daily sessions, frequency, age of onset, marijuana quantity, concentrates quantity, and edibles quantity. Two items did not show clean factor loadings and were therefore removed. The final 39-item version of the DFAQ-CU is provided in the S1 File.

Using Cronbach’s alpha as a measure of internal consistency, the frequency factor was found to demonstrate excellent reliability, the age of onset and marijuana quantity factors showed good reliability, and the daily sessions and cannabis concentrate factors demonstrated acceptable reliability. The daily sessions factor showed the lowest reliability, which likely relates to the fact that it only contains two items. Moreover, we originally created those items to assess frequency of use. Finally, since the edibles quantity factor only contains one item, internal consistency could not be computed. Based on these results, we suggest a more cautious approach when using the daily sessions and edibles quantity factors. In contrast, the remaining four factors appear to be reliable indicators of frequency, age of onset, and quantity of marijuana and concentrates used.

Most of the factors were found to demonstrate high convergent validity. The exceptions were the daily sessions, marijuana quantity and cannabis concentrate quantity factors, which demonstrated adequate convergent validity, and the edible quantity factor, which demonstrated sub-par convergent validity. This may however reflect the fact the MSHQ and TLFB only measure quantity using number or size of joints. The lack of existing measures for assessing quantity of cannabis in other forms makes it difficult to determine whether the DFAQ-CU quantity factors are valid indicators of quantity of cannabis used.

Scores on the various measures of cannabis use disorders and problematic cannabis use (i.e., CAST, CUDIT-R, CUPIT, MSI-X) were used to assess the DFAQ-CU’s predictive validity. The results revealed that the DFAQ-CU’s frequency factor showed excellent predictive validity, the daily sessions factor demonstrated good predictive validity, and the age of onset, concentrates quantity, and edibles quantity factors showed acceptable predictive validity. In contrast, the marijuana quantity factor showed suboptimal predictive validity. This pattern of results may reflect the fact that frequency of cannabis is often an indicator of cannabis use disorders and cannabis-related problems, while age of onset and quantity of cannabis used are typically disregarded in the assessment of cannabis-related problems [34]. Therefore, the lower predictive validity of the age of onset and quantity factors may simply reflect the fact that these aspects of cannabis use are not as strongly related to cannabis use disorders and cannabis-related problems, rather than problems with validity per se.

Finally, scores on the AUDIT, which measures harmful use of alcohol, were used to assess the divergent validity of the DFAQ-CU factors. The daily sessions, concentrate quantity, and marijuana quantity factors demonstrated excellent divergent validity. While the frequency, age of onset, and edible quantity factors showed small but significant relationships with AUDIT scores, for the frequency factor, these correlations were consistently smaller than those with the MSHQ, TLFB, CAST, CUDIT-R, CUPIT, and MSI-X and they likely reflect the fact that cannabis users tend to use more alcohol than non-users [35, 36]. While it is common to use alcohol measures to assess discriminant validity of cannabis use/abuse measures [37, 38] future research should attempt to assess the discriminant validity of the DFAQ-CU by using measures of constructs unrelated to alcohol and substance use.

In addition to the 22 core items, the DFAQ-CU contains items that are used to establish skip logic and further screen or characterize the sample. These items include a follow-up item to be administered to individuals who indicate that they used cannabis ‘today,’ that assesses how high the participant feels. This item can be used to screen out individuals who are currently experiencing acute effects of cannabis or to explore those effects. The DFAQ-CU also includes items to assess the amount of time participants have been using cannabis at the frequency reported, the average frequency they used cannabis before that time, and the total number of years they have used cannabis. These items can be used to better understand respondents’ history of cannabis use, to screen out individuals who have been using cannabis for only short periods of time, and/or to compare long-term cannabis users with short-term users.

The DFAQ-CU also assess the primary and secondary types of cannabis used (marijuana, concentrates, edibles). While these items are primarily used to determine which quantity items to administer, researchers can also use these items to characterize their sample and to explore putative differential effects in individuals who primarily use concentrates or edibles rather than marijuana. The DFAQ-CU also contains items to assess medical cannabis use, and, when applicable, the medical conditions cannabis is used for and the percentage of time that cannabis is used for recreational rather than medical purposes. These items will allow researchers to characterize their sample and to explore potential differences in medical vs. recreational vs. combined users.

In appreciation of apprehensions about the rising levels of THC in cannabis [39] and the subsequent need to investigate whether high THC products intensify harms, the DFAQ-CU also includes two items designed to assess typical levels of THC in marijuana and concentrates consumed. We recognize that measuring the average THC levels of marijuana and concentrates is currently problematic because much of the cannabis sold is not labeled or may be mislabeled. For this reason, participants are instructed to leave these items blank if they do not know the typical THC levels of the products they use. Nevertheless, tightening regulations on the labeling of legal cannabis may help to reduce these problems in the future, thereby allowing researchers to examine the effects of high vs. low THC.

The measurement of quantity of cannabis use is complicated by numerous factors, including the different potencies, methods of ingestion, types of cannabis, and the social nature of cannabis use [40]. We have attempted to overcome these hurdles by assessing typical THC levels in cannabis used and different methods of ingestion. Our quantity items are also stated to encourage respondents to consider only personal use. Further, quantities of different types of cannabis are separately measured by the DFAQ-CU. Moreover, to our knowledge the DFAQ-CU is the first inventory to include actual pictures of marijuana in joint, bud, and loose-leaf forms to facilitate the identification of the quantity of marijuana typically used. While we have not overcome all of the obstacles to measuring quantity of cannabis use, the DFAQ-CU represents a substantial improvement.

The present study is limited by the use of a college student sample that comprised predominantly female recreational cannabis users and by the lack of any biological indicator of cannabis use. The use of a predominantly female sample is problematic because typically males are more likely to use cannabis, are more likely to become dependent upon cannabis, and to initiate cannabis use at a younger age [41]. However, there is evidence that the gender gap in cannabis use is decreasing [42, 43]. Also college students are a commonly targeted demographic for examining the chronic and residual effects of cannabis, in part because young adults are the most common group to use cannabis [3]. Further, given the rapidly changing legal landscape surrounding cannabis, it will be important for future research to replicate these results in both legal and illicit cannabis users and to contrast findings across these groups to ensure generalizability. Future research should also examine test-retest reliability, to replicate the study using broader community samples, and to further validate the frequency and quantity factors using biological indicators of cannabis use.

Despite these limitations, the results of the present study indicate that the DFAQ-CU is a psychometrically sound inventory for measuring daily sessions, frequency, age of onset, and quantity of marijuana, concentrates, and edibles used. By offering researchers a valid and reliable means of assessing these dimensions of cannabis consumption as well as a resource for better screening and characterizing their samples, the DFAQ-CU should help to improve and intensify research efforts focused on examining the effects of cannabis consumption on humans.

## Supporting information

S1 File DFAQ-CU inventory. (DOCX)

S2 File Dataset. (SAV)
