# Supplementary material for: Measuring cannabis consumption: Psychometric properties of the Daily Sessions, Frequency, Age of Onset, and Quantity of Cannabis Use Inventory (DFAQ-CU)
Source: PLoS One. 2017 May 26;12(5):e0178194. doi: 10.1371/journal.pone.0178194 (PMC5446174; doi:10.1371/journal.pone.0178194)
Supplement: S1 File — (DOCX) [file pone.0178194.s001.docx]

**DFAQ-CU Inventory**

**Instructions:** Please read each of the following questions and mark the response alternative that best describes your use of cannabis. *Note that the term cannabis is being used to refer to marijuana, cannabis concentrates, and cannabis-infused edibles.*

1. Have you ever used cannabis?

0 = No

1 = Yes

**If response = 0 then skip to end of questionnaire*

2. Which of the following best captures when you last used cannabis?

1 = over a year ago

2 = 9 – 12 months ago

3 = 6 – 9 months ago

4 = 3 – 6 months ago

5 = 1 – 3 months ago

6 = less than 1 month ago

7 = last week

8 = this week

9 = yesterday

10 = today*

11 = I am currently high*

**If response = 10 (today) or 11 (I am currently high) then answer 2b below*

2b. How high are you right now?

0 = I am not at all high

1 = I am a little bit high

2 = I am moderately high

3 = I am very high

4 = I am extremely high

3. Which of the following best captures the average frequency you currently use cannabis?

0 = I do not use cannabis

1 = less than once a year

2 = once a year

3 = once every 3-6 months (2-4 times/yr))

4 = once every 2 months (6 times/yr)

5 = once a month (12 times/yr)

6 = 2 – 3 times a month

7 = once a week

8 = twice a week

9 = 3 – 4 times a week

10 = 5 – 6 times a week

11 = once a day

12 = more than once a day

4. Which of the following best captures how long you have been using cannabis **at this frequency?**

1 = less than 1 month

2 = 1 – 3 months

3 = 3 – 6 months

4 = 6 – 9 months

5 = 9 – 12 months

6 = 1 – 2 years

7 = 2 – 3 years

8 = 3 – 5 years

9 = 5 – 10 years

10 = 10 – 15 years

11 = 15 – 20 years

12 = more than 20 years

5. Before the period of time you indicated above, how frequently did you use cannabis?

0 = I did not use cannabis

1 = less than once a year

2 = once a year

3 = once every 3-6 months (2-4 times/yr.)

4 = once every 2 months (6 times/yr.)

5 = once a month

6 = 2 – 3 times a month

7 = once a week

8 = twice a week

9 = 3 – 4 times a week

10 = 5 – 6 times a week

11 = once a day

12 = more than once a day

6. How many days of the past week did you use cannabis?

0 = 0 days

1 = 1 day

2 = 2 days

3 = 3 days

4 = 4 days

5 = 5 days

6 = 6 days

7 = 7 days

7. Approximately how many days of the past month did you use cannabis? ____________

8. Which of the following best captures the number of times you have used cannabis in your entire life?

1 = 1 – 5 times in my life

2 = 6 – 10 times in my life

3 = 11 – 50 times in my life

4 = 51 –100 times in my life

5 = 101 – 500 times in my life

6 = 501 – 1000 times in my life

7 = 1001 – 2000 times in my life

8 = 2001 – 5000 times in my life

9 = 5001 – 10,000 times in my life

10 = More than 10,000 times in my life

9. Which of the following best captures your pattern of cannabis use throughout the week?

0 = I do not use cannabis at all

1 = I only use cannabis on weekends

2 = I only use cannabis on weekdays

3 = I use cannabis on weekends and weekdays

10. How many hours after waking up do you typically first use cannabis?

0 = I do not use cannabis at all

1 = 12 – 18 hours after waking up

2 = 9 – 12 hours after waking up

3 = 6 – 9 hours after waking up

4 = 3 – 6 hours after waking up

5 = 1 – 3 hours after waking up

6 = within 1 hour of waking up

7 = within ½ hour of waking up

8 = immediately upon waking up

11. How many times a day, on a typical weekday, do you use cannabis? ____________

12. How many times a day, on a typical weekend, do you use cannabis? ____________

13. What is the primary method you use to ingest cannabis?

0 = I do not use cannabis

1 = Joints

2 = Blunts (cigar sized joints)

3 = Hand pipe

4 = Bong (water pipe)

5 = Hookah

6 = Vaporizer (e.g., Volcano, Vape pen)

7 = Edibles

8 = Other _______________________

14. Which of the following other methods to ingest cannabis do you use **regularly** (at least 25% of the time use you cannabis)? [Mark all that apply]

0 = None

1 = Joints

2 = Blunts (cigar sized joints)

3 = Hand pipe

4 = Bong (water pipe)

5 = Hookah

6 = Vaporizer (e.g., Volcano, Vape pen)

7 = Edibles

8 = Other _______________________

15. What is the primary form of cannabis you use?

0 = None****

A = Marijuana***

B = Concentrates (e.g., Oil, Wax, Shatter, Butane Hash Oil, Dabs)**

C = Edibles*

D = Other____________________

16. What other forms of cannabis do you use **regularly** (at least 25% of the time you use cannabis)? [Mark all that apply]

0 = None****

A = Marijuana***

B = Concentrates (e.g., Oil, Wax, Shatter, Butane Hash Oil, Dabs)**

C = Edibles*

D = Other____________________

*****If response to questions 15 and 16 = 0 (None) then skip to question 29*

****If responses to questions 15 or 16 = A (Marijuana) then answer questions 17-21*

***If responses to question 15 or 16 = B (Concentrates) then answer questions 22-26*

**If responses to question 15 or 16 = C (Edibles) then answer question 27*

*Note: If you use more than one form of cannabis then complete all of the associated questions listed above.***If responses to questions 15 or 16 = A (Marijuana) then answer questions 17-21 below.*

Please use the image below to refer to various quantities of marijuana. The image is not to scale; the dollar bill is included to help provide size perspective.

*
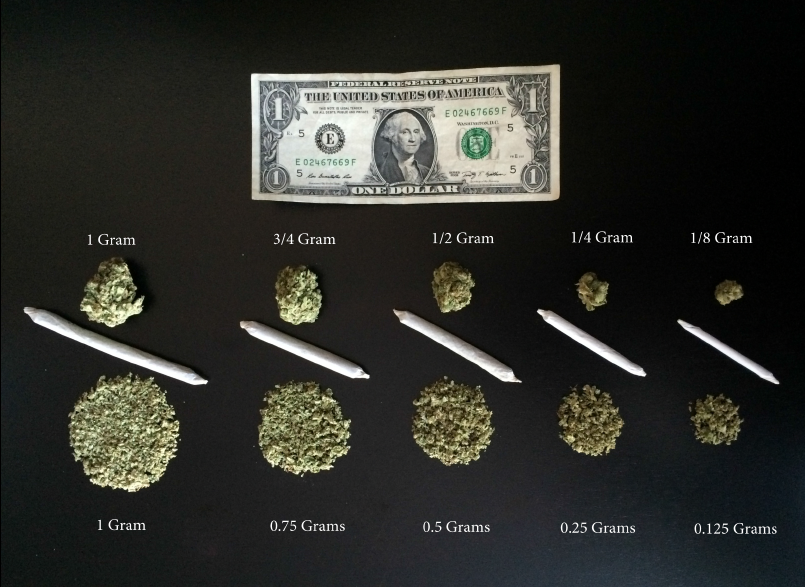
*

For questions 17 to 19 below, clearly indicate the number of grams of marijuana you use with a number between 0 – 100. Do NOT include other forms of cannabis you may use (such as concentrates). You may use up to 3 decimals to indicate amounts under 1 gram.

Note: 1/8 of a gram = 0.125 grams, ¼ of a gram = 0.25 grams, ½ of a gram = 0.5 grams, ¾ of a gram = 0.75 grams. 1/8 of a ounce = 3.5 grams, ¼ of an ounce = 7 grams, ½ ounce = 14 grams, 1 ounce = 28 grams

17. In a typical session, how much marijuana do you personally use? ______________________

18. On a typical day you use marijuana, how much do you personally use? _________________

19. In a typical week you use marijuana, how much marijuana do you personally use? ________

20. On a typical day you use marijuana, how many sessions do you have? __________________

21. What is the average THC content of the marijuana you typically use? Leave blank if you do not know.

1 = 0 – 4%

2 = 5 – 9%

3 = 10 – 14%

4 = 15 – 19%

5 = 20 – 24%

6 = 25 – 30%

7 = greater than 30%

***If response to questions 15 or 16 = B (Concentrates) then answer questions 22-26 below*

22. In a typical session you use cannabis concentrates, how many hits do you personally take? __

23. On a typical day you use cannabis concentrates, how many hits do you personally take? ____

24. How many hits of cannabis concentrates did you personally take yesterday? _____________

25. On a typical day you use cannabis concentrates, how many sessions do you have? _________

26. What is the average THC content of the concentrates you typically use? Leave blank if you do not know.

1 = 0 – 9%

2 = 10 – 19%

3 = 20 – 29%

4 = 30 – 39%

5 = 40 – 49%

6 = 50 – 59%

7 = 60 – 69%

8 = 70 – 79%

9 = 80 – 90%

10 = greater than 90%

***If response to questions 15 or 16 = C (Edibles) then answer question 27 below*

27. When you eat edibles how many milligrams of THC do you personally ingest in a typical

session? ___________

28. What is your current age? ___________

29. How many years in total have you used cannabis? ___________

30. How old were you when you FIRST tried cannabis? ___________

31. Has there been any time in your life when you used cannabis regularly (2 or more times per month for 6 months or longer)?

0 = No

1 = Yes*

**If response = 1 (Yes) then answer questions 31b and 31c below*

31b. How old were you when you FIRST STARTED using cannabis regularly (2 or more

times/month)? ___________

31c. Has there been any time in your life when you used cannabis on a daily or near daily basis for 6 months or longer?

0 = No

1 = Yes*

**If response = 1 (Yes) then answer question 31ci below*

31ci. How old were you when you FIRST STARTED using cannabis on a daily or

near daily basis? ___________

32. Which of the following best captures the average frequency that you used cannabis before the age of 16?

0 = more than once a day

1 = once a day

2 = 5 – 6 times a week

3 = 3 – 4 times a week

4 = twice a week

5 = once a week

6 = 2 – 3 times a month

7 = once a month

8 = once every 2 months (6 times/yr.)

9 = once every 3-6 months (2-4 times/yr.)

10 = once a year

11 = less than once a year

12 = never

33. Do you have a physician’s recommendation to use cannabis for medicinal purposes?

0 = No

1 = Yes*

2 = Yes, but I use it for both medicinal and recreational purposes*

**If response = 1 or 2 (Yes) then answer questions 33b and 33c*

33b. Which medical condition(s) do you use cannabis for?

________________________________________________________________________

33c. What percentage of the time do you use cannabis for recreational (rather than

medicinal) purposes? ________________

**DFAQ-CU Scoring**

Daily Sessions Items: 20, 25

Frequency Items: 2, 3, 6, 7, 8, 9, 10, 11, 12

Age of Onset Items: 30, 31b, 31ci, 32

Marijuana Quantity Items: 17, 18, 19

Concentrate Quantity Items: 22, 23, 24,

Edibles Quantity Item: 27

*Note: Standardize (z-transform) scores prior to calculating the mean of each of the 6 factors (daily sessions, frequency, age of onset, marijuana quantity, concentrate quantity, edibles quantity).*

Screening/Characterization Items: 1, 2b, 4, 5, 13, 14, 15, 16, 21, 26, 28, 29, 31, 31c, 33, 33b, 33c

Contact us at: DFAQCU@gmail.com if you would like a Qualtrics version of the DFAQ-CU shared to your Qualtrics account.
